# Exogenous and Endogeneous Disialosyl Ganglioside GD1b Induces Apoptosis of MCF-7 Human Breast Cancer Cells

**DOI:** 10.3390/ijms17050652

**Published:** 2016-04-30

**Authors:** Sun-Hyung Ha, Ji-Min Lee, Kyung-Min Kwon, Choong-Hwan Kwak, Fukushi Abekura, Jun-Young Park, Seung-Hak Cho, Kichoon Lee, Young-Chae Chang, Young-Choon Lee, Hee-Jung Choi, Tae-Wook Chung, Ki-Tae Ha, Hyeun-Wook Chang, Cheorl-Ho Kim

**Affiliations:** 1 Molecular and Cellular Glycobiology Unit, Department of Biological Sciences, SungKyunKwan University, 300 Chunchun-Dong, Jangan-Gu, Suwon City, Kyunggi-Do 440-746, Korea; sunspring5@naver.com (S.-H.H.); jmlee@daum.net (J.-M.L.); muscaria80@naver.com (K.-M.K.); hahaaaa@nate.com (C.-H.K.); pokusa6@hotmail.com (F.A.); wnsdud2057@naver.com (J.-Y.P.); 2 Research Institute, Davinch-K Co., Ltd., B1603-3, 606, Seobusaet-gil, Geumcheon-gu, Seoul 153-719, Korea; 3 Division of Enteric Diseases, Center for Infectious Diseases Research, Korea National Institute of Health, Heungdeok-gu, Cheongju 363-951, Korea; skcho38@hotmail.com; 4 Functional Genomics Laboratory, Department of Animal Sciences, the Ohio State University, 2029 Fyffe Court, Columbus, OH 43210, USA; lee.2626@osu.edu; 5 Research Institute of Biomedical Engineering and Department of Medicine, Catholic University of Daegu School of Medicine, Daegu 705-718, Korea; ycchang@cu.ac.kr; 6 Faculty of Medicinal Biotechnology, Dong-A University, Busan 604-714, Korea; yclee@dau.ac.kr; 7 Division of Applied Medicine, School of Korean Medicine, Pusan National University, Yangsan City, Gyeongsangnam-Do 626-870, Korea; pure0917@hanmail.net (H.-J.C.); chungtw@hanmail.net (T.-W.C.); haggis@pnu.ac.kr (K.-T.H.); 8 College of Pharmacy, Yeungnam University, Gyeongsan 712-749, Korea; hwchang@yu.ac.kr; 9 Department of Medical Device Management and Research, Samsung Advanced Institute for Health Sciences & Technology (SAIHST), Seoul 06351, Korea

**Keywords:** disialyl-ganglioside GD1b, human breast cancer MCF-7 cells, apoptosis, caspase, human β1,3-galactosyltransferase-2 (GD1b synthase, Gal-T2)

## Abstract

Gangliosides have been known to play a role in the regulation of apoptosis in cancer cells. This study has employed disialyl-ganglioside GD1b to apoptosis in human breast cancer MCF-7 cells using exogenous treatment of the cells with GD1b and endogenous expression of GD1b in MCF-7 cells. First, apoptosis in MCF-7 cells was observed after treatment of GD1b. Treatment of MCF-7 cells with GD1b reduced cell growth rates in a dose and time dependent manner during GD1b treatment, as determined by XTT assay. Among the various gangliosides, GD1b specifically induced apoptosis of the MCF-7 cells. Flow cytometry and immunofluorescence assays showed that GD1b specifically induces apoptosis in the MCF-7 cells with Annexin V binding for apoptotic actions in early stage and propidium iodide (PI) staining the nucleus of the MCF-7 cells. Treatment of MCF-7 cells with GD1b activated apoptotic molecules such as processed forms of caspase-8, -7 and PARP (Poly(ADP-ribose) polymerase), without any change in the expression of mitochondria-mediated apoptosis molecules such as Bax and Bcl-2. Second, to investigate the effect of endogenously produced GD1b on the regulation of cell function, UDP-gal: β1,3-galactosyltransferase-2 (GD1b synthase, Gal-T2) gene has been transfected into the MCF-7 cells. Using the GD1b synthase-transfectants, apoptosis-related signal proteins linked to phenotype changes were examined. Similar to the exogenous GD1b treatment, the cell growth of the GD1b synthase gene-transfectants was significantly suppressed compared with the vector-transfectant cell lines and transfection activated the apoptotic molecules such as processed forms of caspase-8, -7 and PARP, but not the levels of expression of Bax and Bcl-2. GD1b-induced apoptosis was blocked by caspase inhibitor, Z-VAD. Therefore, taken together, it was concluded that GD1b could play an important role in the regulation of breast cancer apoptosis.

## 1. Introduction

Gangliosides are glycosphingolipids composed of a series of ceramide-linked oligosaccharides with one or more sialic acids (*N*-acetylneuraminic acid) linked on the sugar chain. They are important in various cellular functions including cellular interaction, differentiation, transmembrane signaling, tumor progression, receptor functions and apoptosis [1,2,3]. With regard to induction of cellular apoptosis, our group has recently reported the apoptotic effect of gangliosides GM3 on colon cancer cells [4]. In addition, effect of disialosylganglioside GD3 on the induction apoptosis has been demonstrated on leukemic Jurkat T cells [5], neuronal cells [6], vascular endothelial cells [7] and vascular smooth muscle cells [8].

GD3 has been confirmed as a cell death effector, contributing to the mitochondria-dependent apoptosome activation and subsequent apoptosis triggered by death ligands [9]. Similarly, GD3 was reported to induce apoptosis of human colon cancer Colo-205 cells and human breast carcinoma SKBR3 cells [10]. For the disialosylganglioside GD2, it has been recently reported that GD2-specific antibodies suppressed the cell growth and induced apoptosis of small cell lung cancer cell lines (SCLC) [11]. Despite the studies showing the proapoptotic properties of disialosylgangliosides in tumor cells, few studies have focused on the proapoptotic function of disialosylganglioside GD1b. Recently, Ma *et al.*, [12] reported that GD1b induced apoptosis in human breast carcinoma SKBR3 cells, but the role of GD1b in apoptosis induction of the tumor cells has been remained unknown.

Apoptosis has been well defined with regard to cell death for development and tissue homeostasis in multi-cellular organisms [13,14]. Apoptotic cells are accompanied with several morphological and molecular changes through the induction or activation of various apoptotic molecules such as caspase, bax, bcl-2 and PARP (Poly(ADP-ribose) polymerase) [10,15]. There are two functional caspase groups, initiator caspases (caspase-2, -8, -9 and -10) and effector capases (capase-3, -6 and -7) [16]. Functionally, initiator caspases are known to activate the downstream effector caspases, which then cleave PARP as a substrate [17,18]. Therefore, tumor therapy has regarded the induction of apoptosis as the most important anti-cancer mechanism [19]. Breast cancer is one of the major cancers in women and is known to be highly metastatic [20]. Among human breast cancer cells, it has been reported that the MCF-7 and SK-BR-3 cells have limited migration whereas the MDA-MB-468 cells are more migratory than the first two [21]. The breast cancer MDA-MB-231 cells are highly migratory [21,22,23]. In breast cancer cells, the abundant gangliosides are GM3, GM2, GM1, and GD1a in MDA-MB-231 and MCF-7 [24]. In particular, GM3 was the major ganglioside in MDA-MB-231 cells, while the most predominant ganglioside is GM1 in MCF-7 cells, lacking GD1b. To understand the relationship between the apoptotic potentials and GD1b in the above cells, therefore, we have examined the effect of GD1b on MCF-7 cell apoptosis.

**Figure 1:**
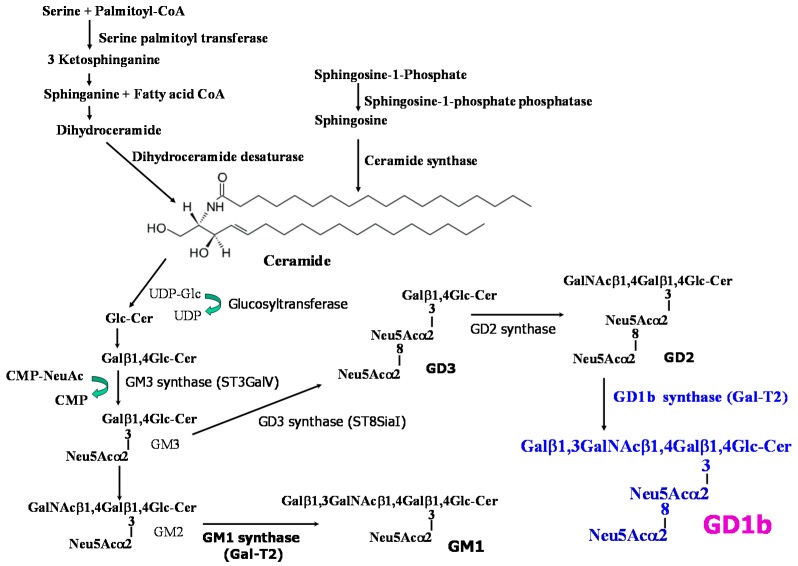
Structures of gangliosides and biosynthetic pathway of disialo GD1b.

In this study, therefore, we demonstrated that exogenous and endogenous GD1b is involved in regulation of MCF-7 cell growth. Following exogenous GD1b treatment, changes in cell growth rate and the degree of apoptosis were detected in a dose- and time-dependent manner in MCF-7 cells. In addition, GD1b specifically decreased cell growth and induced apoptosis whereas other gangliosides do not, as observed by flow cytometry and immunofluorescence assay and detection of apoptosis-related signal molecules. Gangliosides are *de novo* synthesized through ER-Golgi pathway from ceramide by serial addition of sugar residues in animal cells (Figure 1).To take deep insight into the action mechanism of GD1b, GD1b synthase gene has been transfected to the MCF-7 cells. Endogenously overexpressed GD1b suppressed growth and induced apoptosis of MCF-7 cells, as similarly observed in exogenous treatment of GD1b. Taken together, GD1b has been regarded to be a novel therapeutic candidate drug to treat the human breast cancers.

## 2. Results

### 2.1. Suppression of Cell Growth by GD1b

**Figure 2:**
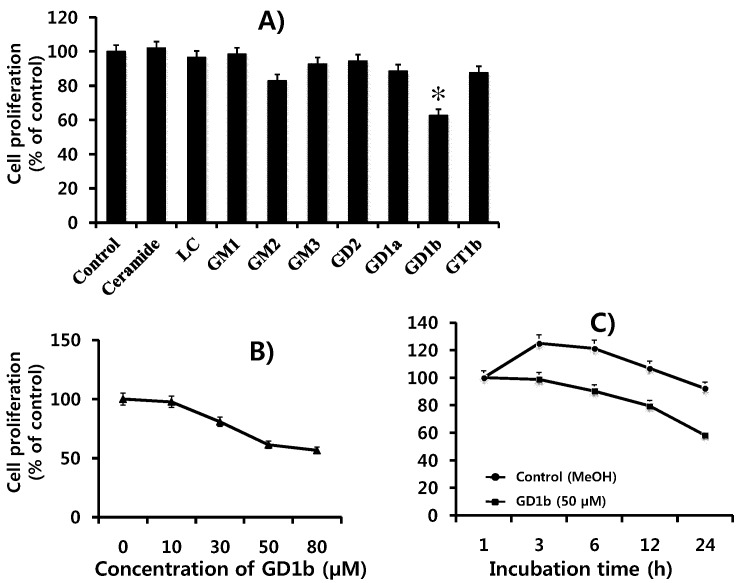
Effect of various gangliosides on MCF-7 cell growth. (**A**) The cytotoxicity of various gangliosides on the MCF-7 cells has been examined using an XTT kit for cell growth assay. The cultured cells (approximately 1 × 10^4^ cells) in 96-well microplates (volume, 100 μL/well) for 24 h with various gangliosides were checked for the cytotoxicity; (**B**,**C**) The cytotoxicity of the GD1b on the MCF-7 cells was evaluated using an XTT cell proliferation assay kit. Cells were exposed to GD1b at various concentrations (0 to 80 μM) and also incubated with time course (1, 3, 6, 12 and 24 h). Control was treated with methanol (8 μL/100 μL) only. The absorbance at a wavelength of 490 nm was then measured using a digitalized ELISA reader (Molecular Devices, Sunnyvale, CA, USA). Data are reported as the percentage change in comparison with the control group, which were arbitrarily assigned as 100% viability. Data represent five experiments (means ± SD). * *p* < 0.01 *vs.* control.

The effects of various gangliosides on MCF-7 cell growth were examined. As shown in Figure 2A, the resulting survival curve shows that only cells treated with GD1b showed a cytotoxic effect whereas other gangliosides or ceramide did not have any effect on MCF-7 cells. Then, we examined the effects of GD1b on cell growth of MCF-7 cells with various concentrations using the XTT assay. When MCF-7 cells were treated with various concentrations of GD1b for 24 h, GD1b rapidly decreased the growth of MCF-7 cells in a dose-dependent manner as seen in Figure 2B. The growth of MCF-7 cells treated with 50 µM of GD1b was significantly decreased in a time-dependent manner (Figure 2C). Therefore, it was finding that GD1b inhibits the growth of MCF-7 cells.

### 2.2. Induction of Apoptosis by GD1b in MCF-7 Cells

**Figure 3:**
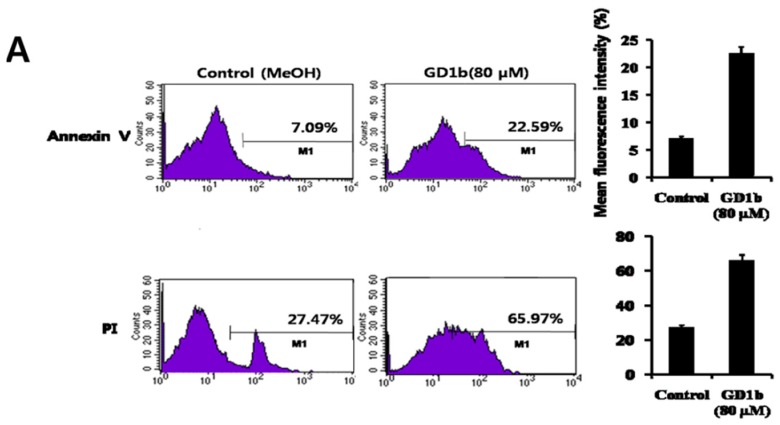

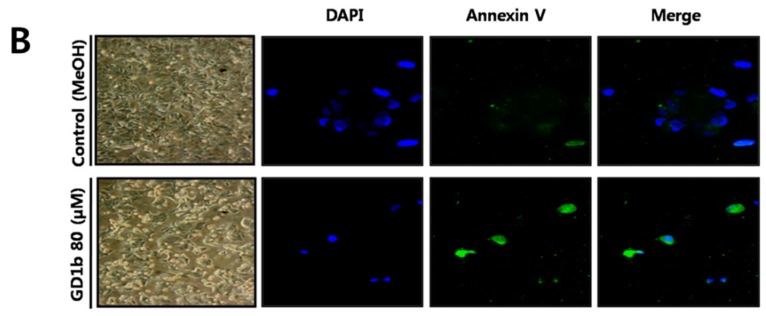
Apoptosis of MCF-7 cells by GD1b treatment. (**A**) Double staining of the MCF-7 cells treated GD1b using annexin V and PI. Control (left) was treated with 80 µL/mL of methanol and right phannal was treated with 80 µM of GD1b for 24 h and stained with FITC-conjugated Annexin V (**top**) and PI (**bottom**) and then analyzed by flow cytometry; (**B**) Effect of GD1b on MCF-7 cells morphological changes (left). Morphological changes of control cells and cells treated GD1b were observed under a phase-contrast microscope with 100×. Apoptosis of MCF-7 cells treated with GD1b was stained with DAPI and Annexin V. Control (upon) was treated with 80 µL/mL of methanol. Cells sown in the lower pannel was treated with 80 µM of GD1b for 24 h and stained with FITC-conjugated Annexin V and DAPI and then analyzed by immunocytostaining (magnification 63×).

To clarify the induction of apoptosis during the growth suppression of GD1b treated MCF-7 cells, cells were double stained with Annexin V (FITC) and PI, since Annexin V is a cell membrane marker specific for early stage apoptosis and PI can enter to the nucleus resulting from the cell membrane permeability changes at the later stage of apoptosis [25]. As shown in Figure 3A, when MCF-7 cells treated with GD1b were stained with Annexin V and PI at 24 h after the addition of GD1b, the induction of apoptosis was observed. MCF-7 cells treated with GD1b were positively stained with Annexin V (22.59%) and with PI (65.97%) 24 h after the GD1b treatment with the cells, indicating the GD1b induces apoptotic cell death of the MCF-7 cells. In Figure 3B, morphological changes in control cells and MCF-7 cells treated with GD1b were observed under a confocal microscope. Following 24 h treatment with GD1b, significant differences in MCF-7 cell shape was observed between the control and the GD1b (80 µM)-treated cells. Cell-to-cell contact of the MCF-7 cells treated with 80 µM GD1b was inhibited and detachment of the MCF-7 cells from the substratum with cytoplasmic rounding was also observed. Furthermore, the treated cells also showed marked shrinkage of the cytoplasm and formation of spaces between cells, whereas control cells showed no change. Apoptotic MCF-7 cells treated with GD1b were positively stained with DAPI and Annexin V, whereas the control group, which was treated with 80 μL/mL of methanol, was not stained. As shown in Figure 3B (lower panel), MCF-7 cells treated with GD1b were stained with a higher intensity by Annexin V. When the two stains were merged, MCF-7 cells treated with GD1b showed a pattern with Annexin V dominating the staining, whereas the control were almost only stained with DAPI. Therefore, the result suggests that GD1b induced effectively apoptosis of MCF-7 cells.

### 2.3. Induction of Apoptosis Related-Molecules by GD1b

**Figure 4:**
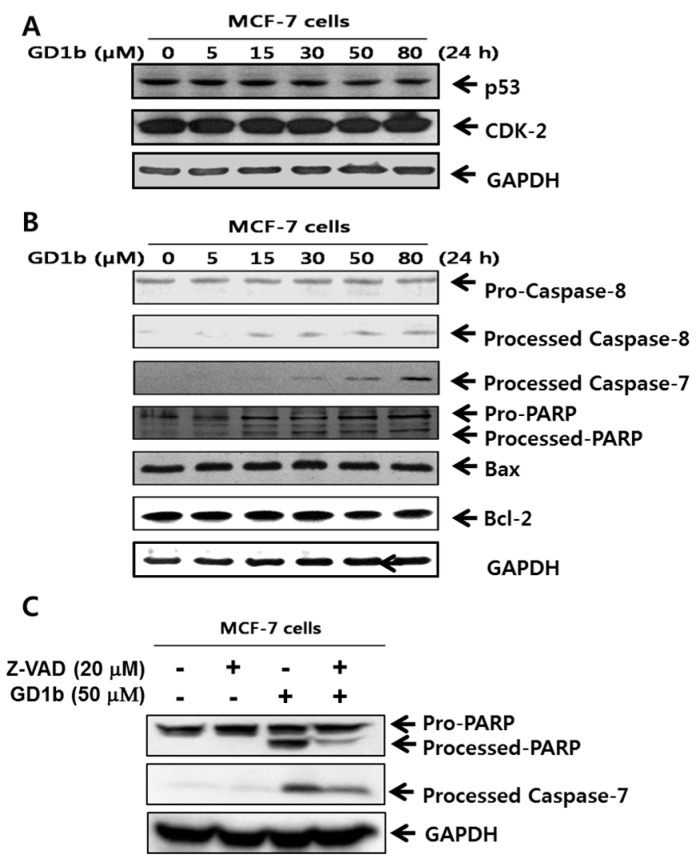
Effects of GD1b treatment on cell cycle arrest and apoptosis. The lysates containing equal protein content were separated by SDS-PAGE and analyzed by western blotting. Expression of cell cycle arrest- (**A**) and apoptosis- (**B**) related molecules were examined of protein extracts of MCF-7 cells treated without (negative control with methanol) or with various concentrations of GD1b for 24 h. MCF-7 cells were treated with 50 µM GD1b for 24 h in the presence or absence of 20 µM Z-VAD and then analyzed by immunoblotting (**C**) with antibody PARP and capase-7. GAPDH was used as the internal control.

To demonstrate the activity of cell apoptosis proteins of MCF-7 cells treated with GD1b, immunoblot analysis was performed to determine caspase cleavage. It was shown that pro-caspase 8 was cleaved to its active form processed caspase-8. The active caspase-8 then activates downstream pro-caspase-7. For various apoptotic situations, cleavage of PARP in GD1b-treated MCF-7 cells is shown in Figure 4. The PARP is known to be activated by caspase-7. These results suggested the involvement of Caspase-8, -7 and PARP activation during apoptosis of the MCF-7 cells occurred at concentrations dose-dependent manner by GD1b treatment. Interestingly, treatment of MCF-7 cells with GD1b did not change in expression of mitochondria-mediated apoptosis molecular such as Bax and Bcl-2. To further confirm that GD1b induces apoptosis that depends on the caspase cascade, MCF-7 cells were treated with pan-caspases inhibitor, 20 µM Z-VAD and 50 µM GD1b, and then PARP and caspase-7 were analyzed by immunoblotting (Figure 4C). Levels of cleaved caspase-7 and PARP were increased in the presence of Z-VAD with GD1b-treated MCF-7 cells. Overall, GD1b induces caspase-dependent apoptosis. Therefore, the result suggests that GD1b induces the apoptosis by caspase-specific manner, but not mitochondria-mediated apoptosis in MCF-7 cells.

### 2.4. Expression of Gangliosides by Transfection of GD1b Synthase

The nucleotide sequence of the human β1,3-galactosyltransferase-2 gene (GM1 and GD1b synthase; *Gal-T2*, GeneBank accession number Y15014) was initially used to generate the Gal-T2 reverse antisense primer and perform a BLAST analysis. As a result of Blast analysis, high sequence identities were observed from the GD1b synthase gene in MCF-7 cells. Since the GD1b synthase gene was rarely expressed in MCF-7 cells in our preliminary experiment by TLC, the DNA fragment carrying the GD1b synthase gene was obtained by gene amplification using RT-PCR, from which the sequence of GD1b synthase gene was found to be 1269 bp long. A DNA fragment encoding for amino acids 422 of GD1b synthase was inserted into the HindIII/BamHI site of pcDNA3.1 vector.

**Figure 5:**
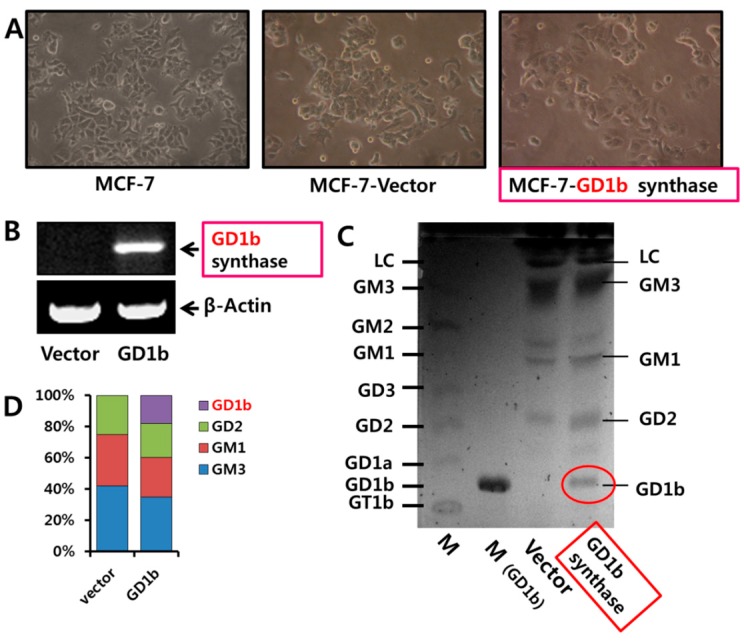
Establishment of GD1b synthase-transfected MCF-7 cell line. (**A**) Effect of GD1b synthase transfection on MCF-7 cells morphological changes. Morphological changes of MCF-7, vector- (pcDNA 3.1 vector) and GD1b (pcDNA 3.1 vector/GD1b synthase)-transfectant cells were observed under a phase-contrast microscope with 100×; (**B**) Expression levels of GD1b synthase of vector- and GD1b-transfectant cells were analyzed by RT-PCR. β-Actin was used as the internal control; (**C**) Gangliosides contents in vector- and GD1b-trnasfectant cells were analyzed by TLC; (**D**) Comparison of ganglioside contents in Mock (vector-transfected) MCF-7 cells and GD1b synthase-transfected MCF-7 cells. Percentage of each ganglioside was measured by densitometry with total gangliosides of each lane as 100%.

After the transfection of MCF-7 cells with expression vector (pcDNA3.1) and the pcDNA3.1/GD1b synthase which carries the GD1b synthase gene, the Vector- or GD1b synthase-transfectant cells were established. Figure 5A showed the morphological changes in vector or GD1b synthase-transfectants cells. MCF-7 cells and vector-transfectant cells showed sharp pointed types in morphology, though the GD1b synthase-transfectant cells changed into a round shape. In addition, GD1b synthase-transfectants cells had a marked shrinkage of cytoplasm, indicating cell death.

Expression levels of GD1b synthase mRNA was highly expressed in GD1b synthase-transfectant cells compared with the vector-transfectant cells, examined by RT-PCR (Figure 5B). The overexpression of GD1b (Figure 5C) showed a marked increase in the expression of GD1b in the GD1b synthase-transfectants cells compared with the vector-transfectant cells. However, expression of GM1 decreased in GD1b synthase-transfectant cells. Being compared the composition of gangliosides by densitometry (Figure 5D) GD1b levels in the vector 18% greater in GD1b synthase-transfectant cells. However, GM1 levels was 25% less in GD1b synthase-transfectant cells. Therefore, the result suggests that Vector- or GD1b synthase-transfectants cells were established.

### 2.5. Suppression of Cell Growth by GD1b Overexpression

**Figure 6:**
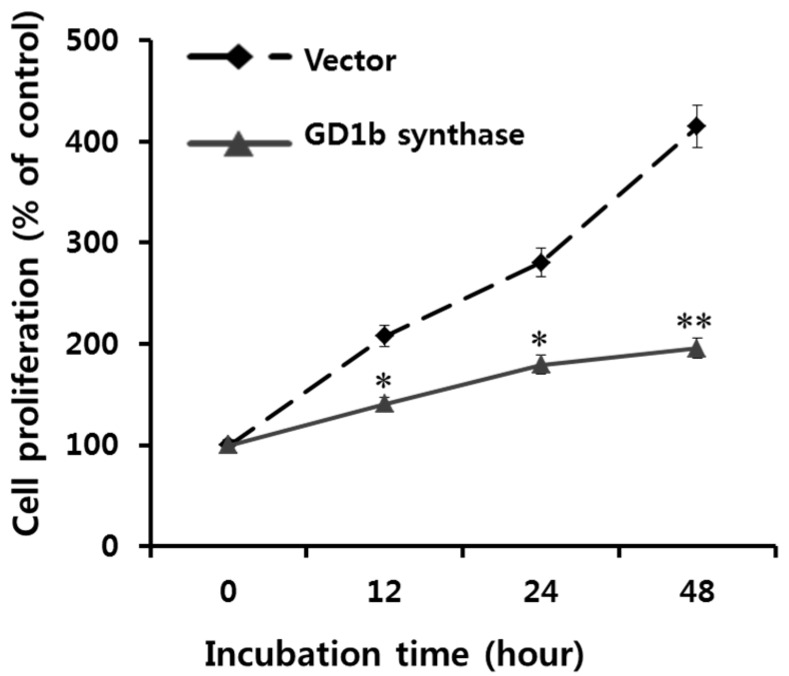
Effects of GD1b overexpression on the cell growth. The growth of vector- and GD1b-transfectant cells was evaluated using an XTT cell proliferation assay kit. Vector- and GD1b-transfectant cells were serum (+) incubated for 0, 12, 24 and 48 h. All data are reported as the percentage change in comparison with the control group, which were arbitrarily assigned 100% viability. Data represent five experiments (means ± sd). * *p* < 0.01 and ** *p* < 0.001 *vs.* vector (mock) transfectants, at each incubation time.

The effects of GD1b overexpression on cell proliferation were examined using the XTT assay. As shown in Figure 6, the GD1b synthase-transfectant cells showed a markedly reduced cell proliferation rate in comparison with vector-transfectant cells. These results suggest that endogenously overexpression of GD1b suppressed growth of MCF-7 cells, similar to what was observed with the exogenous treatment of GD1b.

### 2.6. Induction of Apoptosis Related Molecules by GD1b Overexpression

**Figure 7:**
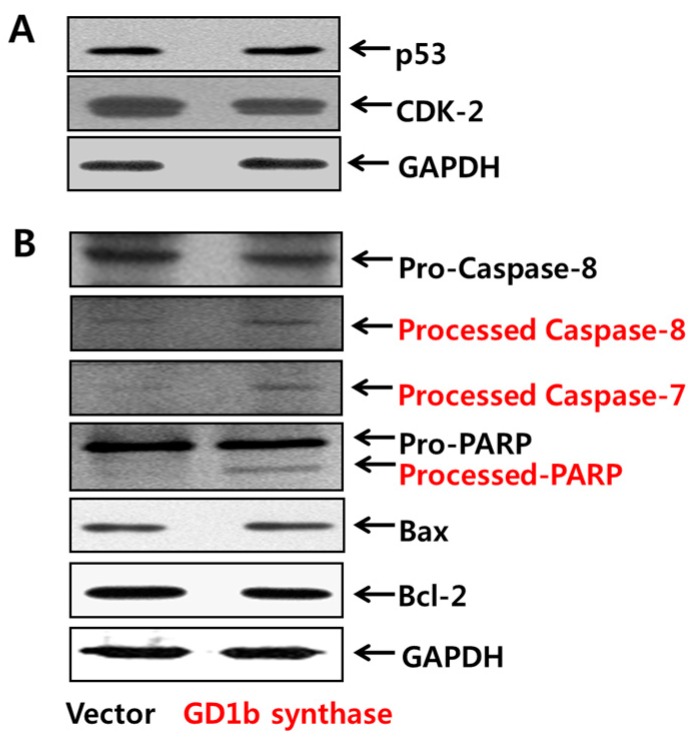
Effects of GD1b overexpression on cell cycle arrest and apoptosis. The cell lysates with equal protein content were subjected to SDS-PAGE and the separated proteins were analyzed by Western blotting using each antibody. Expression of cell cycle arrest- (**A**) and apoptosis- (**B**) related molecules were examined of protein extracts of Vector- and GD1b-transfectant cells. GAPDH was used as the internal control.

Western blot analysis was performed to demonstrate the activity of cell cycle arrest- and apoptosis-related molecules of transfectant cells. First, GD1b synthase-transfectant cells did not have any changes in the expression of cell cycle arrest-related molecules such as p-53 and CDK-2 compared with vector-transfectant cells (Figure 7A). Second was the examination of the caspase activation in vector- or GD1b synthase-transfectant cells (Figure 7B). During apoptotic cell death, Pro-caspase-8 is catalytically cleaved to caspase-8, its active form. The active caspase-8 further cleaves downstream pro-caspase-7 to active caspase-7 by its cascade mechanism. Pro-capase-7 activated downstream PARP. These results suggested the involvement of Caspase-8, -7 and PARP activation during apoptosis of the GD1b synthase-transfectant cells. However, GD1b synthase-transfectant cells did not have any changes in the expression of mitochondria-mediated apoptosis molecular such as Bax and Bcl-2. Therefore, the result suggests that overexpression of GD1b induces the apoptosis by caspase-specific manner but not mitochondria-mediated apoptosis molecules, as observed in exogenous treatment of GD1b.

## 3. Discussion

Apoptosis can be mediated by three major signaling pathways including death receptor-, mitochondria- and Endoplasmic Reticulum (ER)-mediated pathways [26]. First, plasma membrane death receptors triggering external apoptosis signaling belong to the tumor necrosis factor (TNF)-receptor super-family. TNF-R promotes apoptosis via the adaptor proteins TNFR-associated death domain (TRADD) and the activation of caspase-8, where procaspase-8 is proteolytically activated to the active caspase-8 state, which then activates downstream effector caspases [27]. Second, in mitochondria-mediated apoptosis, the mitochondrial membrane induces the release of Cytochrome c, which are regulated by pro-apoptotic (Bax, Bak, Bad, Bid, Bik, Bcl-xs) and anti-apoptotic (Bcl-2, -xL, -w) proteins in caspase-dependent and -independent apoptosis pathways [28]. Finally, the ER is another regulation site for cell death. In the ER-mediated pathway, caspase-12 on the cytoplasmic side of the ER acts as an initiator caspase [29].

It is known that glycosphingolipids are directly linked to cellular recognition, signal transduction, differentiation, apoptosis, growth, attachment, adhesion, migration, metastasis and angiogenesis [5,6,7,8]. Gangliosides, complex glycosphingolipids containing *N*-acetylneuraminic acid and attached to a ceramide, are synthesized in the Golgi and components to plasma membranes and other organelles [30] that modulate cell signal transduction events. Gangliosides appear that they concentrate in lipid rafts. There have been a number of reports on the modulation of cell growth and or differentiation by glycosphingolipids [31,32,33]. Many of the studies were performed by adding exogenous gangliosides into the culture medium of cell lines under investigation. There are many reports that gangliosides induced proliferation inhibition of cancer cells [34]. Among them, Ma *et al.* reported that GD3 and GD1b could play an important role in the regulation of SK-BR-3 human breast cancer cell death [12]. GD3 and GD1b are disialosyl-ganglioside containing two *N*-acetylneuraminic acids and GD1b is synthesized by β-1,3-galactosyltransferase or GD1b synthase (galactosyltransferase II) through catalyzing the addition of a second sialic acid residue to one of its immediate precursor GD2. GD1b is one of the breast cancer associated gangliosides. In a preliminary result, treatment of the cells with GD1b induced apoptosis on SKBR-3 and MDA-MB-231 cells. Interestingly, it was evident that MCF-7 cells were highly sensitive to apoptosis induction when GD1b was treated. In this result, GD1b down-regulated growth in MCF-7 cells (Figure 2A). When MCF-7 cells were treated with GD1b under various (concentration and time) conditions (Figure 2B,C), the results showed that GD1b treatment decreased cell growth of MCF-7 cells in a dose- and time-dependent manner. Previously, ceramide induced growth inhibition in prostate cancer cells [35], but MCF-7 cells treated with ceramide showed no growth inhibition in this study, indicating a specific property in GD1b had a growth inhibition effect.

Through several biochemical experiments, it was shown that GD1b induces cell growth regulation in MCF-7 cells. MCF-7 cells treated with GD1b were stained with higher intensity FITC-conjugated Annexin V and PI than normal MCF-7 cells and apoptotic morphological changes were observed by microscope and immunocytostaining. MCF-7 cells treated with GD1b showed marked shrinkage of cytoplasm, formation of spaces between cells, FITC-conjugated, and annexin V stained, whereas control cells showed no change (Figure 3). Taken together, GD1b has apoptotic actions in early stage and the nucleus of MCF-7 cells. Our findings indicated that exogenous GD1b plays an apoptotic role in MCF-7 cells.

Because survival pathways contribute to the opposite concept of cancer cells to cancer therapy, we examined that exogenous GD1b was no changed with cell cycle arrest-related proteins (Figure 4A). Another interesting finding of the present study was that the exogenous GD1b effectively induces the altered expression of the proteins, which are known to be related to apoptosis. This was partly shown through the induction of apoptosis signal. Recently, it was reported that MCF-7 cells were devoid of caspase-3 and the caspase-3 deficiency was responsible for atypical PARP cleavage [36,37]. Initiator caspases-8 and PARP (down signal molecule) was shown to be induced by GD1b in this study. As modulators of mitochondria-mediated apoptosis pathway, Bcl-2 and Bax family are related to the anti-apoptotic and pro-apoptotic effects, respectively [38,39]. The treatment of MCF-7 cells with GD1b did not alter the expressions of Bax and Bcl-2 in this study. Therefore, it was suggested that caspase-mediated apoptotic factors are involved in the GD1b-stimulated apoptosis of MCF-7 cells, but not by mitochondria-mediated pathway, as shown in Figure 4.

The GD1b is synthesized by β1,3-galactosyltransferase-2 (GD1b synthase, Gal-T2). To examine the function of endogenously synthesized GD1b synthase in MCF-7 cells, we constructed pcDNA3.1 expressing vector- and pcDNA3.1-GD1b-transfectant cell lines. The GD1b-transfectant cells overexpressed GD1b synthase and consequently ganglioside GD1b. Although GD3 were not detected on TLC, GD1b was detected in large amounts, suggesting that vector- or GD1b-transfectant cells were successfully established (Figure 5C). The cell number of MCF-7 cells treated exogenously with GD1b was decreased to under the initial stage of 100%. When MCF-7 cells were treated with 30, 50 and 80 µM GD1b for 24 h, the growth of MCF-7 cells was decreased to 78%, 62% and 58%, respectively (Figure 2), suggesting that the growth of MCF-7 cells treated with GD1b was time-dependently decreased. Endogenously overexpressed GD1b significantly suppressed growth of MCF-7 cells, when compared to the vector (mock) transfectants at each incubation time (Figure 6). However, in the endogenous GD1b synthesis assay, the anti-cell proliferative activity of GD1b was not much high compared to the exogenous GD1b treatment with the MCF-7 cells (Figure 6), showing that the growth of MCF-7 cells transfected with the GD1b synthase gene was increased slightly rather than the initial stage of growth. Therefore, the difference between the exogenous and endogenous effects of GD1b on the anti-proliferation of MCF-7 cells is interesting in its biological function. For the discrepancy between the results of exogenous GD1b treatment and endogenous GD1b synthesis assay, there is the issue of endogenously expressed gangliosides are possibly interacted with cellular molecules to function as secondary messengers and building machinery in cellular organelle, as our previous indicated that endogenously expressed GD3 assembled into apoB-containing lipoproteins [40]. The GM3 and GD3 gangliosides in each of the transfected cells were found in the cytosols to associate with apolipoproteins, but not in the medium only. In the report, it was clearly suggested that the cultured medium of GD3 synthase-transfected cells are enriched with triglyceride-containing apoB, confirming an increased secretion of triglyceride-enriched apoB. In this study, for example, intracellularly produced GD1b might be localized in those cellular compartments or used as substantial regulator after the ER-Golgi biosynthetic pathway. Therefore, it was indicated that GD1b inhibits the growth of MCF-7 cells and GD1b induce the apoptosis in MCF-7. The fate and function of the gangliosides, which are intracellularly biosynthesized, are potential topic of the glycobiological research field, as discussed earlier [41,42]. Taken together, these results have clearly indicated that the GD1b, regardless if endogenously produced or applied exogenously, inhibits the growth of MCF-7 cells. In addition, the overexpression of GD1b activated the apoptotic molecules such as process of caspase-8, -7 and PARP. However, this overexpression of GD1b in MCF-7 cells did not affect the expression of mitochondria-mediated apoptosis molecules such as Bax and Bcl-2, as observed in exogenous treatment of GD1b, too.

The human breast cancer MCF-7 cells has estrogen receptor (ER) and EGF receptors, depending on estrogen and EGF for growth, and is not invasive, while human breast cancer MDA-MB-231 cells and SK-BR-3 cells are aggressive and estrogen-independent during growth [24]. These basal type breast cancer cells of MDA-MB-231 cells and SK-BR-3 cells are known as triple-negative breast cancers for ER, progesterone receptor and erbB2 with poor prognosis. Among both, especially, MDA-MB231 is a relative highly metastatic and invasive breast cancer model [43] and thus targeted therapeutic approaches have been tried [44,45,46]. Although the two cell types of SK-BR-3 and MDA-MB-231 cells are ER-negative, however, the function of gangliosides is controversial at this stage. For example, overexpressed ganglioside GD3 enhances cellular proliferation and migration capacity of MDA-MB-231 cells [47], while gangliosides such as GD3 and GD1b were reported to induce rather apoptosis in SK-BR3 cells [12]. For the ER-positive human breast cancer cell line, the ganglioside content of MCF-7 cells was reported to be higher than MDA-MB-231 cells and membrane-inserted ganglioside GM3 of MCF-7 cells blocked EGF-stimulated growth [24]. As the ganglioside analysis showed that GD1b is expressed from the GD1b synthase-transfected MCF-7 cells, but not in MCF-7 cells (Figure 5C). Although there is still covered for each specific ganglioside effect in the ER-negative and ER-positive breast cancer cells, GD1b of ER-positive MCF-7 cells might be interacted with membrane proteins to induce apoptosis. The efficiency of molecular targeted therapies for ER-positive breast cancer cells to inhibit growth and induce apoptosis is limited due to the basic resistance such as mutation. Therefore, the ER-positive breast cancer cells can benefit from GD1b-based targeted therapies together with hormone therapy, chemical products and Herceptin administration [46]. The present results suggest that GD1b ganglioside could be effective therapeutic target in ER-positive breast cancers. The present results suggest that targeting both GD1b ganglioside and apoptotic molecules would lead to a therapeutic outcome. However, further studies on which intracellular and membrane proteins are interacted with GD1b ganglioside for the specific apoptosis are required.

## 4. Material and Methods

### 4.1. Cells and Materials

The human breast cancer MCF-7 cells were obtained from the American Type Culture Collection (Manassas, VA, USA) and kept in the laboratory. Z-VAD-FMK (pancaspase inhibitor) was purchased from Calbiochem (EMD Biosciences, San Diego, CA, USA). Cells were maintained in Dulbecco’s modified Eagle’s medium (DMEM, Gibco, Karlsruhe, Germany) supplemented with 10% fatal bovine serum (FBS) obtained from Welgene Co. (Daegu, Korea), 100 g/mL streptomycin (Sigma, St. Louis, MO, USA) and incubated at 37 °C in a humidified atmosphere incubator containing 5% CO_2_ in air.

### 4.2. XTT Proliferation Assay

Cell proliferation and growth assay was monitored using a commercially available cell proliferation kit II (XTT, Boehringer Mannheim, Germany). In brief, the cultured MCF-7 cells with DMEM were further subcultured into 96-well culture microplates (SPL Lifesciences, Pocheon, Gyunggi, Korea) in 100 μL of DMEM at a density of 10 × 10^3^ cells per each well in humidified incubator. The growth cells were then allowed to attach for 2.5 h. After 24 h of further incubation at the same condition, the medium was discarded and replaced with 100 μL of new DMEM with gangliosides (0, 10, 30, 50 and 80 µM). GD1b rapidly decreased the growth of MCF-7 cells in a dose-dependent manner as seen in Figure 1B. The cells treated with each ganglioside were incubated for 24 h in a 37 °C incubator under a humidified atmosphere of 5% CO_2_. After the incubation time for 24 h, each DMEM medium was discarded from the cells and the cells were washed with DMEM medium and phosphor buffered saline (PBS). 50 μL of XTT test solution, what was mixed with two reagents (50:1 ratio of sodium 3V-(1-(phenyl-aminocarbonyl)-3,4-tetrazolium)-bis(4-methoxy-6-nitro) benzenesulfonic acid hydrate and *N*-methyl dibenzopyrazine methyl sulfate) of XTT-labeling reagent (5 mL) and electron coupling reagent (100 μL), was added to each well, depending on the supplier’s guideline of protocol. After incubation in a 37 °C and 5% CO_2_ incubator for 4 h, ELISA assay was performed to measure the absorbance at 490 nm wavelength on an ELISA reader (Molecular Devices, Sunnyvale, CA, USA). All experimental measurements were independently carried out to determine the effects of gangliosides on cell growth from 3 independent experiments.

### 4.3. Flow Cytometry Assay

Apoptosis was identified by flow cytometry with Annexin V-FITC/PI staining (BD Pharmingen, San Diego, CA, USA), as described by the manufacturer’s mannuals. Briefly, MCF-7 cells were treated with each ganglioside, as described above, and then the cells were centrifuged for 5 min at 3000 rpm. To discard the cell medium, the supernatant was removed and the remaining cells were added with 100 μL of binding buffer and 3 µL of Annexin V-FITC. At the condition shielded from light, the cells were incubated at room temperature for 15 min. 100 μL of binding buffer and 3 µL of PI were added to the cells. MCF-7 cells treated with 80 µM of methanol were used as a negative control. The fluorescence of 10,000 events per sample was analyzed using a Becton- Dickinson FACScan (Becton Dickinson, Franklin Lakes, NJ, USA).

### 4.4. Immunofluorescence

In the absence or presence of 80 µM GD1b, MCF-7 cells were incubated with 3 μg/mL of Annexin V-FITC for 15 min. Aliquots of cells were prepared and fixed on coverslides. Then, coverslides were stained with DNA specific fluorochrome 4,6-diamidino-2-phenylindol (DAPI). The stained cells were analyzed with Confocal laser microscope fluorescence (Olympus, Tokyo, Japan).

### 4.5. Immunoblot Analysis

Equal amount of each protein sample obtained from cell extracts was prepared in SDS sample buffer (1% SDS, 62.5 mM Tris–HCl, pH 6.8, 10% glycerol, 5%-mercaptoethanol, and 0.05% bromphenol blue) was boiled for 5 min. The prepared protein samples were subjected to reducing 8–12% SDS-PAGE. Proteins were separated by SDS-PAGE and electrophoretically transferred to ECL membrane. The ECL membrane was blocked with 5% skim milk in TBS-Tween-20 (2 M Tris–HCl, 4 M NaCl, 0.01% Tween-20) at room temperature for 1 h with two distinct polyclonal rabbit and monoclonal mouse antibodies (1:1000, Santa cruz biotechnology Inc., Paso Robles, CA, USA) directed against the Caspases. Antibodies for PARP, Bcl-2, Bax, CDK2, p53 and GAPDH were purchased from Santa Cruz Biotechnology (Paso Robles, CA, USA). Bio-Rad protein assay was purchased from Bio-Rad (Richmond, CA, USA). After three washes with Tris-Buffered Saline Tween-20 (TBS-T), the blot was incubated with the anti-rabbit and mouse-IgG secondary antibody (1:2000, Santacruz biotechnology Inc., Paso Robles, CA, USA). Proteins which reacted with specific antibodies on electro-transferred membrane were visualized by the protocol supplied from chemiluminescence ECL manufacturer (ECL, Amersham, Piscataway, NJ, USA).

### 4.6. Molecular Cloning of the Human β1,3-Galactosyltransferase-2 Gene (GM1 and GD1b Synthase; Gal-T2) and Reverse Transcription-Polymerase Chain Reaction (RT-PCR)

To isolate the *Gal-T2* gene from MCF-7 cells MCF-7 cells were cultured in DMEM medium and total RNAs was isolated from the MCF-7 cells using the Trizol reagent (Invitrogen, Life Technologies, Carlsbad, CA, USA). Two micrograms of RNAs was subjected to reverse transcription with the random nonamers and cDNA synthesis kit of Takara RNA PCR set (Takara Shuzo, Kyoto, Japan) according to the supplier’s mannuals. GD1b synthase was initially used to generate the reverse antisense primer and perform a BLAST analysis. For the Gal-T2 specific primers, the primers were designed and used (5′-AAGCTTATGCTTCAGTGGAGGAGAAGACACTGCTGC-3′ and 5′-GGATCCCTAATGTAGTTTACGGTGGCGATACCTGCCTGCC-3′) to detect the human β1,3-galactosyltransferase-2 gene (GM1 and GD1b synthase; Gal-T2, GeneBank accession number Y15014). The intensity of the bands obtained from RT-PCR result was estimated using an INV-16M TotalLab software of Chemi- and Fluoro Bio Gel Imaging Analysis System (Davinch-K, Co., Gasan Digital Danji, Seoul, Korea). To confirm the specificity and accuracy of the amplified DNA, the PCR products were subcloned into the pGEM-T vector (Promega, Madison, WI, USA) and sequenced. The finally identified sequence of the isolated clone was identical to the expected cDNA. As a result of Blast analysis using the cloned gene sequence, high sequence identities were observed from the GD1b synthase gene in MCF-7 cells.

### 4.7. Gene Transfection and Selection

Human β1,3-galactosyltransferase-2 (Gal-T2) cDNA were digested by HindIII/BamHI and the fragment containing the coding regions were inserted into the HindIII/BamHI site of pcDNA3.1 to obtain the pcDNA3.1/Gal-T2, respectively. MCF-7 cells used for cDNA transfection were plate in a 60-mm plastic tissue culture plate. Two kinds of cDNA, including the vectors pcDNA3.1 and pcDNA3.1/Gal-T2 were transfected into cells with LipofectAMINETM (Invitrogen, Rockville, MD, USA) according to the supplier’s manual. Among transfectant cells, stably Gal-T2-expressing transfectant cells were selected in the presence of G418 drug (700 μg/mL) (Boehringer Mannheim, Indianapolis, IN, USA) in culture medium.

### 4.8. Extraction of Glycolipids and HPTLC

Cell extracts with chloroform/methanol were directly applied to DEAE Sephadex A-25 columns (Sigma) ion exchange column chromatography. The column was washed to remove neural lipids with chloroform/MeOH/H_2_O. Acidic lipids were eluted by adding chloroform/MeOH and water (3:6:1, *v*/*v*). Then, the acidic glycolipid samples were dissolved in chloroform/MeOH (1:1, *v*/*v*) and applied to chromatographic separation using a DEAE-sephadex A-25 resin (Pharmacia Biotech, Uppsala, Sweden). The column which was bound with non-hydrophobic lipid species was washed with H_2_O to remove them. Sialic acid-bound gangliosides were eluted by adding MeOH and the eluted acidic gangliosides were dried at room temperature under the 12 N ammonia solution treatment for overnight [48]. The lipid fraction was completely dried at 30 °C under speed vacuum and gangliosides were eluted by adding H_2_O. The Sep-Pak C18 cartridge (Millipore, Billerica, MA, USA) was washed with chloroform/MeOH/H_2_O. Then, the acidic lipid samples were applied to sep-pak C18 cartridge column. Gangliosides were eluted by adding chloroform/MeOH (1:1, *v*/*v*). TLC was performed usually with chloroform/methanol/0.2% CaCl_2_ (11:9:2, *v*/*v*), and bands were detected with orcinol spray [49].

### 4.9. Data Analysis

Data are presented as mean ± SD. Statistical comparisons were performed using the Student’s *t* test. A value of *p* < 0.05 was considered to be significant.

## 5. Conclusion

**Figure 8:**
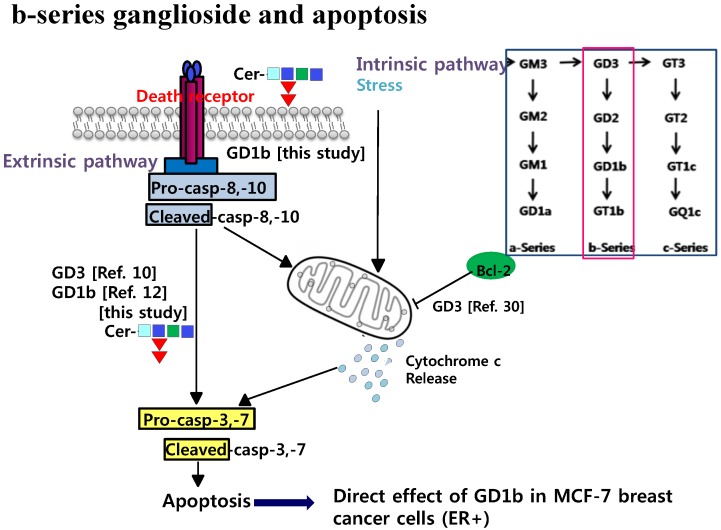
Schematic illustration of b-series gangliosides such as GD1b and GD3 for the apoptotic activities in breast cancer cells. The red square indicates b-series ganglioside during biosynthesis.

In summary, this study showed that GD1b could be effective in the apoptosis regulation of MCF-7 cells. GD1b also was specifically cytotoxic against MCF-7 cells, whereas other gangliosides did not have cytotoxic effects. As shown in Figure 8, the GD1b-mediated apoptosis was also related to caspase pathway. Therefore, we have demonstrated that GD1b-mediated apoptosis activation has an effect on death receptor activation by the dual method of the exogenous treatment of GD1b and the endogenous expression of GD1b in cells. These results are expected to further contribute to the understanding of the anti-cancer activity of GD1b.
